# Differential diagnoses of calcified nodules in pulmonary amyloidosis: A case report

**DOI:** 10.1002/rcr2.1035

**Published:** 2022-09-08

**Authors:** Hiroyuki Miura, Jun Miura, Shinichi Goto, Tomoko Yamamoto

**Affiliations:** ^1^ Department of Thoracic Surgery Akiru Municipal Medical Centre Tokyo Japan; ^2^ Department of Surgery Kyorin University School of Medicine Tokyo Japan; ^3^ Department of Respirology Akiru Municipal Medical Centre Tokyo Japan; ^4^ Department of Pathology Tokyo Women's Medical University Tokyo Japan

**Keywords:** amyloidosis, calcification, pulmonary amyloidosis, pulmonary nodule, transbronchial lung biopsy

## Abstract

Pulmonary amyloidosis should be included in the differential diagnosis of calcified lung nodules, and more careful preparation for bleeding should be taken when performing bronchoscopy. While management does not require aggressive treatment, follow‐up is necessary to monitor for multiple myeloma and malignant lymphoma

## Graphical abstract

Calcified nodules can present as a diagnostic challenge. Pulmonary amyloidosis should be included in the differential diagnosis of calcified lung nodules, and more careful preparation for bleeding should be taken when performing bronchoscopy. Management of nodular pulmonary amyloidosis does not require aggressive treatment, but follow‐up is necessary for complications such as multiple myeloma and malignant lymphoma.

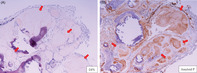

## CLINICAL IMAGE

**FIGURE 1:**
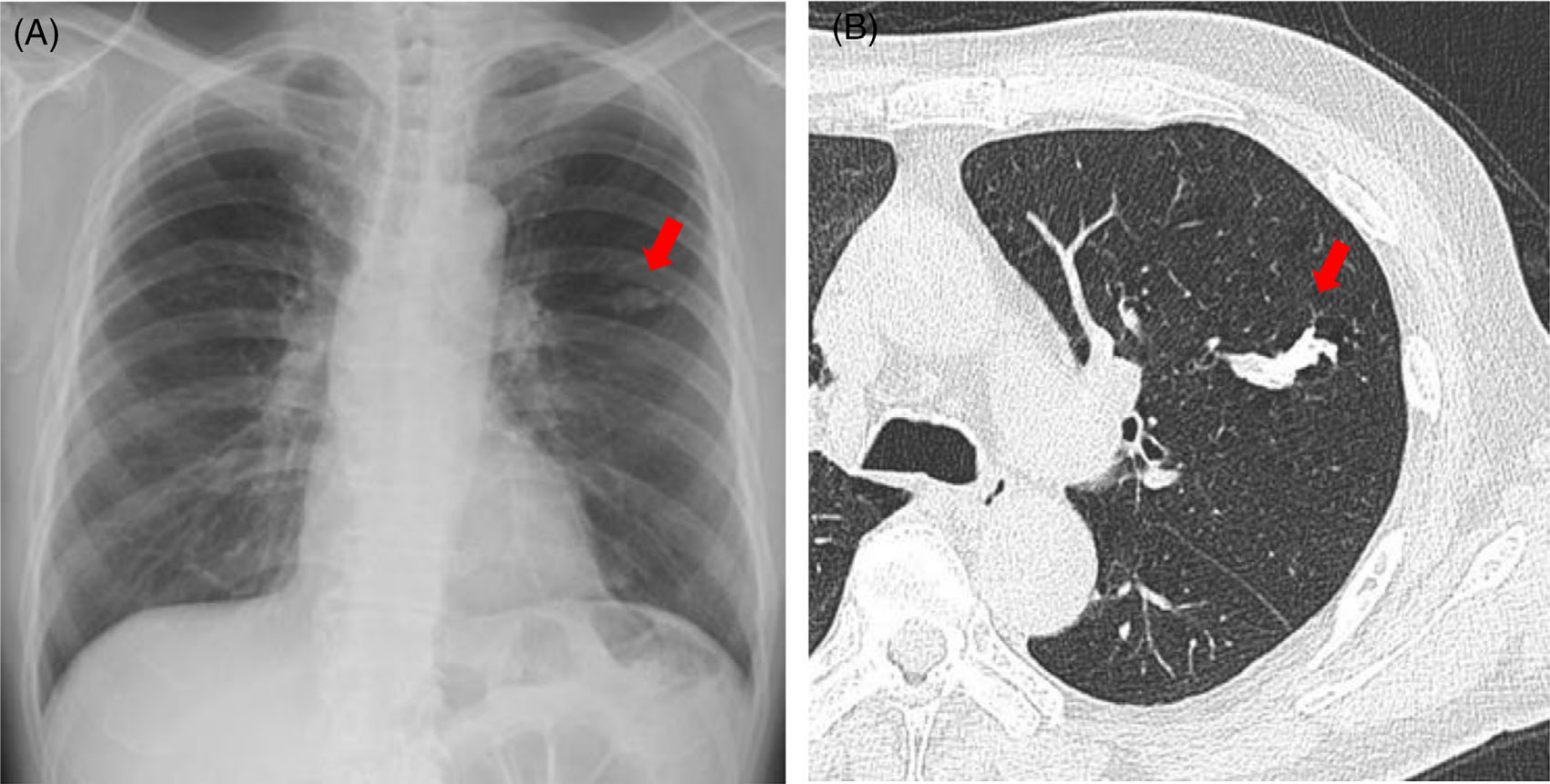
(A) Chest X‐ray showing a spindle‐shaped calcification in the left middle lung field; (B) chest computed tomography (CT) scan showing calcification along the left B3a

**FIGURE 2:**
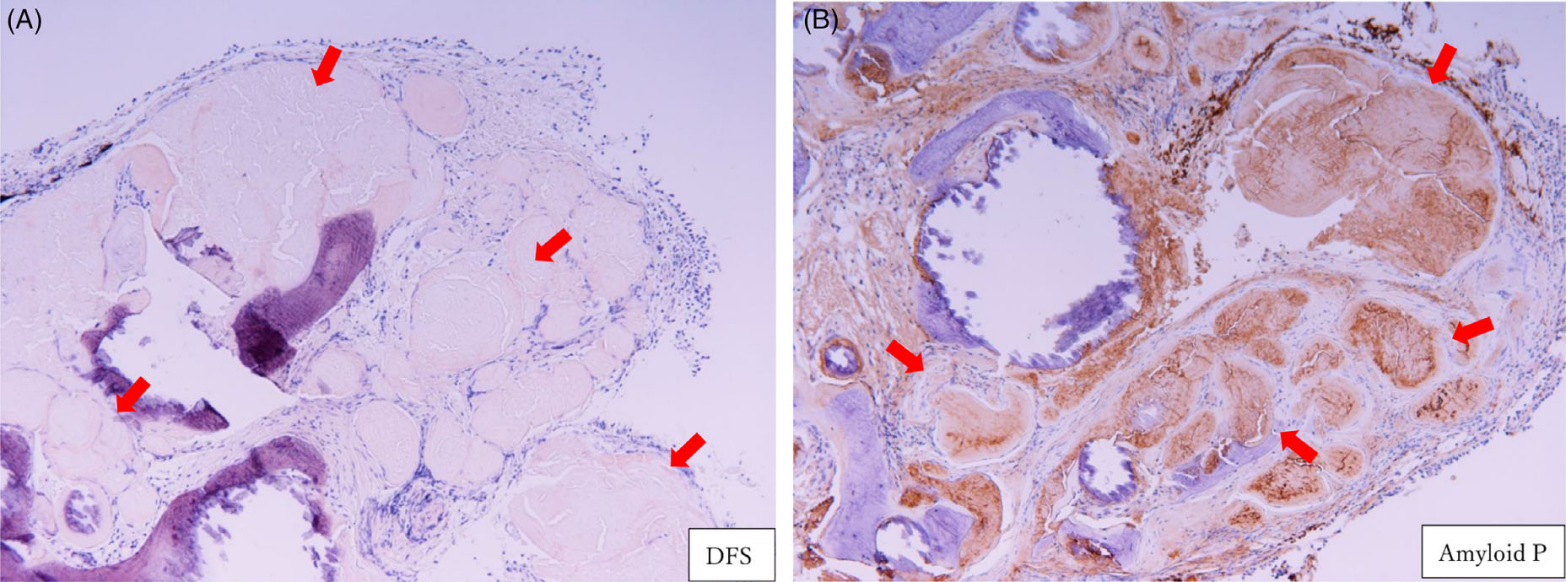
A transbronchial lung biopsy (TBLB) specimen showing direct fast scarlet (DFS) staining‐positive (A) and amyloid P‐positive (B) characteristics

A 76‐year‐old man presented with a chest abnormal shadow during an annual check‐up. His past medical history and family history were unremarkable. He was a current smoker, with 100 pack years. A spindle‐shaped calcification was observed in the left middle lung field (Figure 1A). The chest computed tomography showed calcification along the left B3a (Figure 1B). Serum amyloid A protein level was negative. A transbronchial lung biopsy (TBLB) showed calcified acidophilic structures that stained positively in Congo‐red and direct fast scarlet (DFS) staining. The results, namely, the amyloid A and β2‐microglobulin levels, negative transthyretin staining, and positive amyloid P indicate the AL type (Figure 2A, B).^1^ There was no complication of multiple myeloma or B‐cell lymphoma. The patient was followed up without progression.

Calcification in nodular pulmonary amyloidosis is frequently observed.^1^ Using TBLB to make a diagnosis is technically challenging because the amyloid itself is hard, and the origin of the amyloid is the pulmonary interstitial tissue. It is important to keep in mind the deposition of amyloid in the blood vessels and be prepared for bleeding or air embolism.^2^ Management of nodular pulmonary amyloidosis does not require aggressive treatment, but follow‐up is necessary for complications such as multiple myeloma and malignant lymphoma.

## AUTHOR CONTRIBUTIONS

Hiroyuki Miura and Shinichi Goto were responsible for the conception of the study. Jun Miura was responsible for writing and revising the work. Yamamoto diagnosed this cancer pathologically. All authors contributed to the final version of this manuscript and approved it for publishing.

## CONFLICT OF INTEREST

None declared.

## ETHICS STATEMENT

The authors declare that appropriate written informed consent was obtained for the publication of this manuscript and accompanying images.

## Data Availability

The data that support the findings of this study are available on request from the corresponding author. The data are not publicly available due to privacy or ethical restrictions.
